# Analysis of flow parameters of a Newtonian fluid through a cylindrical collapsible tube

**DOI:** 10.1186/2193-1801-3-566

**Published:** 2014-09-29

**Authors:** Caroline W Kanyiri, Mathew Kinyanjui, Kang’ethe Giterere

**Affiliations:** Department of Pure and Applied Mathematics, Jomo Kenyatta University of Agriculture and Technology, PO Box 62000, Nairobi, Kenya

**Keywords:** Flow parameters, Collapsible tube, Steady flow, Newtonian fluid

## Abstract

In this research study, fluid flow through a cylindrical collapsible tube has been investigated. Of particular interest is the effect of flow parameters on the cross sectional area of a collapsible tube, flow velocity and internal pressure of the fluid. The flow parameters considered are longitudinal tension and volumetric flow rate. The tube is considered collapsible in the transverse direction, taken to be perpendicular to the main flow direction. Collapse happens when external pressure exceeds internal pressure and hence the tube results to a highly noncircular cross sectional area. The fluid flow in consideration is steady and incompressible. Equations governing the flow are non-linear and cannot be solved analytically. Therefore an approximate solution to the equations has been determined numerically. In this case, finite difference method has been used. A computer program has then been used to generate the results which are presented in form of graphs. The results show that the longitudinal tension is directly proportional to both the cross sectional area and internal pressure and inversely proportional to the flow velocity and that change in volumetric flow rate has no effect on the cross sectional area but it is directly proportional to the flow velocity and inversely proportional to the internal pressure.

## 1 Background

Fluid is a type of matter which undergoes continuous deformation when some external force is applied. It is said to undergo deformation if the distance between any two neighboring molecules change. A fluid is said to be Newtonian if it obeys the Newton’s law of viscosity which states that the shear stress is proportional to the velocity gradient. The viscosity does not change with the rate of flow.

A collapsible tube is any tube with sufficiently flexible walls that it can elastically accommodate deformation to a highly noncircular cross section when the external pressure exceeds the internal pressure.

The study of flow through collapsible tubes is of utmost importance in biological studies as well as in industries. Vessel collapse is seen in the veins, such as in the veins of a hand raised above the level of the heart or in the jugular vein when a person is standing upright. In the arteries, collapse occurs when high external pressures are applied, such as when an artery is compressed by a sphygmomanometer cuff during blood pressure measurement. Similarly, in the industry, collapse may be experienced during cementing operations, trapped fluid expansion, or well evacuation, among many others. Most oilfield tubulars also experience collapse.

### 1.1 Literature review

Flow through collapsible tubes has been extensively studied in the laboratory. Pioneering work on collapsible tubes, explain that the veins play an important role in controlling the output of the heart. This control function of the veins is a passive one, and is as a result of their ability to collapse and inflate. Several experimental studies have been done on flexible tubing. For instance Bertram (1986) did an experimental study on collapsible and elastic tubes with finite-length and the upstream and downstream ends held open. He concluded that when the external pressure exceeded the fluid pressure by a sufficiently large amount, the tube buckled non-axi symmetrically, leading to a nonlinear relation between pressure-drop and flow rate.

Bertram et al. (1990) explained that at sufficiently large Reynolds numbers, the system produces self-excited oscillations. Jensen and Heil (2003) also realized that asymmetrically collapsed vessels readily develop flow-induced, self excited oscillations. Physiological examples include wheezing during forced expiration and the development of korottkoff sounds during blood pressure measurement. Earlier works by Luo and Pedley (1998) who investigated the effect of wall inertia on the self-excited oscillations in a collapsible channel flow show that tension-induced instabilities were the main cause of the self-excited oscillations. Makinde (2005) further described the fluid dynamics of a collapsible tube using a mathematical model. He observed that the fluid axial velocity profile was parabolic with maximum value at centerline. He also noted that fluid axial velocity generally decreases with an increase in tube contraction due to the strong influence of the negative transmural pressure owing to marked reduction of rigidity. Marzo et al. (2005) studied three-dimensional collapse of a steady flow through finite-length elastic tubes numerically. Three-dimensional solid elements were used for the elastic wall, allowing the wall thickness to be specified. Previous findings by Hazel and Heil (2003) for thinner-walled tubing were confirmed and also he showed the existence of significant differences if a thick-walled tube is used. Andrew et al. (2008) described the role of venous valves in pressure shielding. A one-dimensional mathematical model of a collapsible tube, with the facility to introduce valves at any position, was used. It was found out that a valve decreased the dynamic pressures applied to a vein when gravity is applied by a considerable amount. Emilie and Patrice (2010) developed a simple and effective numerical physiological tool to help clinicians and researchers in the understanding of flow phenomena. One-dimensional Runge–Kutta discontinuous Galerkin (RK-DG) method coupled with lumped parameter models for the boundary conditions was used. It was noted that the efficiency of muscular calf pump is strongly dependent on the valves pathology and the walking frequency. Eleuterio and Annunziato (2013) formulated a one-dimensional time-dependent non-linear mathematical model for physiological fluid flow in collapsible tubes with discontinuous material properties. He observed that although the solution algorithm dealt with idealized cases, it is uniquely well-suited for assessing the performance of numerical methods intended for simulating more general situations.

This research study has presented a one dimensional mathematical model of fluid flow through collapsible tube. From the Literature review above, a comprehensive study considering the flow parameters such as longitudinal tension and volumetric flow rate and their effects on the cross sectional area of a collapsible tube, flow velocity and internal pressure of fluid in a collapsible tube has not been done. This study therefore aimed at coming up with a more comprehensive model of flow through collapsible tubes thus expanding the understanding of fluid flow through collapsible tubes.

#### 1.1.1 The continuity equation

This equation arises from the fact that matter is neither created nor destroyed. The rate at which mass enters a system is equal to the rate at which mass leaves the system. The differential form for a general continuity equation is given by;
1

where *ρ* is the fluid density and  is the fluid’s velocity.

For incompressible fluid flow, *ρ* is assumed to be a constant and hence equation (1) reduces to;
2

Equation (2) means that the divergence of velocity is zero.

In Cartesian co-ordinate form and considering a one dimensional fluid flow equation (2) is expressed as;
3

The volumetric flow rate Q is given by area multiplied by velocity per unit time, therefore equation (3) becomes;
4

Equation (4) is derived from the fact that mass is always conserved in fluid systems regardless of the pipeline complexity or direction of flow. The volumetric flow rate *Q*is constant but area and velocity of the fluid flow are variable so that if A decreases, *u* increases and vice versa.

#### 1.1.2 Equation of conservation of momentum

The equation of conservation of momentum is derived from Newton’s second law of motion, which states that the time rate change of momentum of a body matter is equal to the net external forces applied to the body. The momentum equation can be expressed as;
5

where R > 0 is a friction factor and g is the gravitational acceleration when the tube is held vertically.

For steady laminar fluid flow, equation (5) is given by,
6

where *s* the peripheral length and *f*_*l*_ is the skin-friction coefficient for laminar flow.

#### 1.1.3 The Tube law

The tube law relates the transmural pressure (internal pressure –external pressure) to the cross-sectional area of a collapsible tube. It is given by,
7

Putting into consideration the longitudinal tension in the tube law equation (7) becomes;
8

Where  and *K*_*PE*_ is the combined stiffness, which represents the overall stiffness of the tube, whether collapsed or distended.

#### 1.1.4 Final set of equations

Making the substitution Q = Au, equation (6) becomes;
9

Which reduces to
10

Thus the final set of equations for a steady laminar fluid flow through a cylindrical collapsible tube are;
11

And
12

## 2 Method of solution

The equations governing the flow problem were written in finite difference form and then reorganized and written in matrix form.

The governing equations describing the steady, incompressible laminar fluid flow through a cylindrical collapsible tube, in finite difference form are given as:
13

where *s*_*i*_ is the peripheral length and is expressed as , *A*_*im*_(*i*) is the area at pressure nodes and is expressed as  and D_*e*_ is the hydraulic diameter expressed as D_*e*_ = 2*πr*.

Equation (13) is derived from equation (11), the derivatives have been replaced by their corresponding finite approximations.

Discretizing equation (12) with central differencing of  yields;
14

The right hand side of equation (14) is linearized using the Taylor expansion of the term  expanded about the point A_*i*_ = c to get equation (15) as follows;
15

Rearranging equation (15) in order to put the like terms together yields;
16

Equation (16) is subject to the following boundary conditions;


where A_0_ is the area at the inlet.

Equation (16) is represented in matrix form and the coefficient matrix is tridiagonal.

The matrix system is of the form  where B is the tridiagonal matrix.

To write the equation in matrix form, let *β* represent 

and *α* represent .

The matrix system of the form  is as shown below;


The tridiagonal matrix B is given by;


And vector R is given by:


### 2.1 Results and discussion

The tridiagonal matrix obtained along with vector R were used to obtain the following graphs using MATLAB program.

#### 2.1.1 Effect of varying Longitudinal Tension on the cross sectional area of the collapsible tube

**Figure 1:**
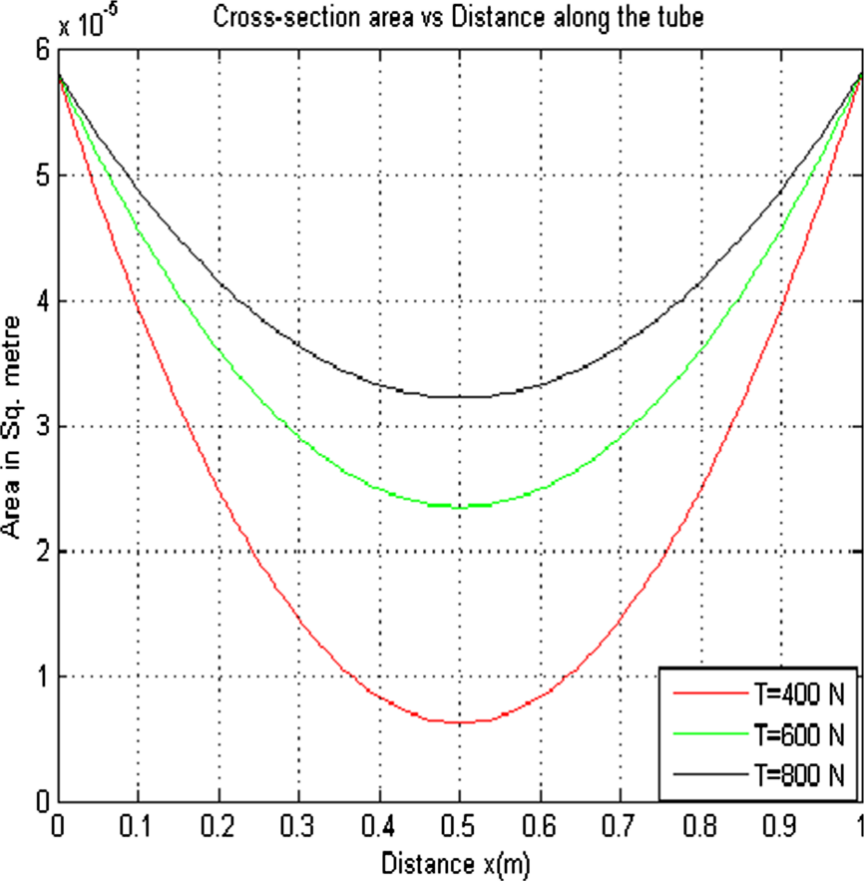
**Cross sectional area versus distance with K**_**PE**_ **= 1.21 × 10** ^**-5**^ **ρ = 1.0 × 10** ^**3**^ **Pe = 4.00 × 10** ^**3**^ **r = 4.3 × 10** ^**-3**^ **Q = 5 × 10** ^**-6**^ **.**

In order to determine the effect of longitudinal tension on the cross sectional area of the collapsible tube, other parameters were held constant while the longitudinal tension was varied. This resulted to three curves that were plotted on the same axes as shown in Figure 1.

From Figure 1 it is observed that when the longitudinal tension was increased from 400 N to 800 N holding the other parameters constant, the cross sectional area increased from 6.206 × 10^-6^ to 3.215 × 10^-5^square meters. This can be explained by the reduction of the tube’s tendency to collapse as the longitudinal tension increases which consequently leads to decrease in collapse hence increase in the cross sectional area.

#### 2.1.2 Effect of varying Longitudinal Tension on the flow velocity

**Figure 2:**
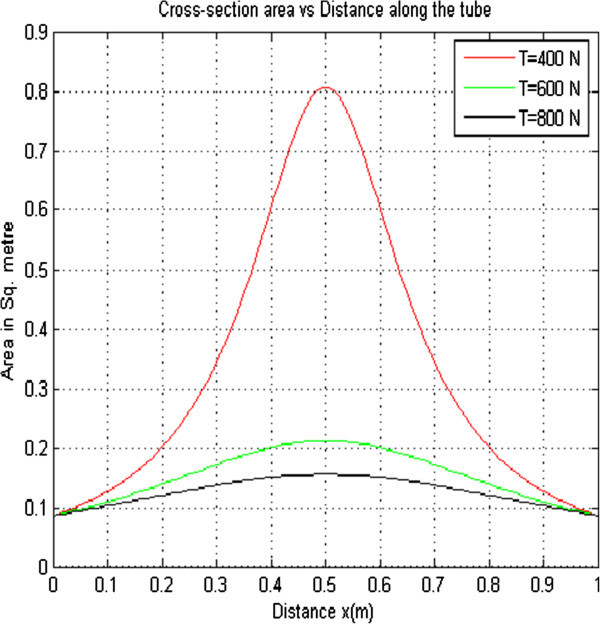
**Flow Velocity versus distance with K**_**PE**_ **= 1.21 × 10** ^**-5**^ **ρ = 1.0 × 10** ^**3**^ **Pe = 4.00 × 10** ^**3**^ **r = 4.3 × 10** ^**-3**^ **Q = 5 × 10** ^**-6**^ **.**

The longitudinal tension was varied while other parameters were held constant. This resulted to three curves that were plotted on the same axes as shown in Figure 2 below.

From Figure 2, it is observed that as the longitudinal tension increases from 400 N to 800 N, the flow velocity decreases from 0.8051 to 0.1555 m/s. The flow velocity decreases when the longitudinal tension increases because of the already increased cross sectional area. This happens in order to maintain a constant discharge.

#### 2.1.3 Effect of varying Longitudinal Tension on the internal pressure

**Figure 3:**
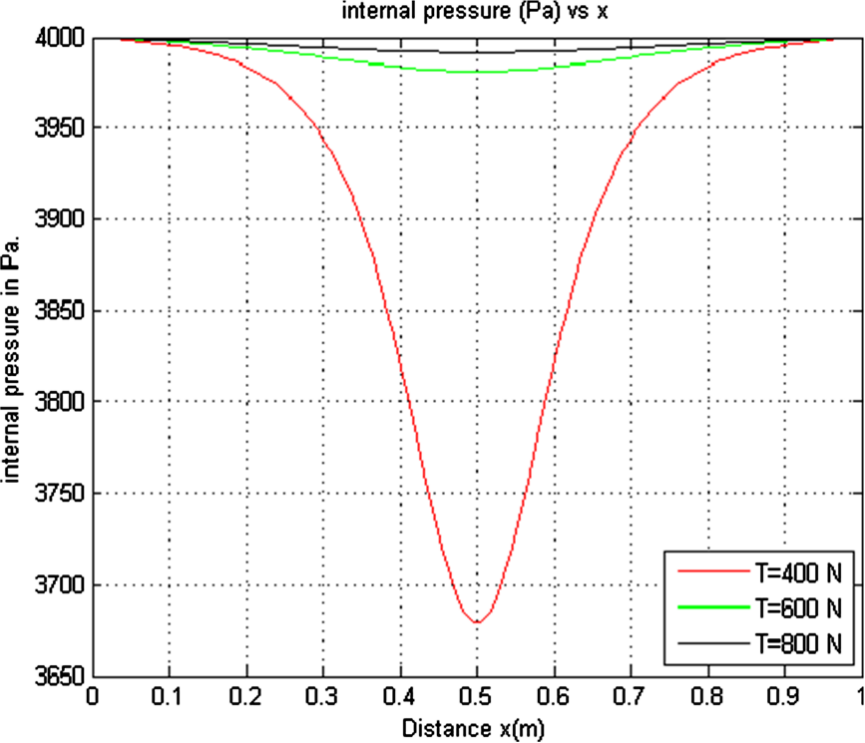
**Internal Pressure versus distance with K**_**PE**_ **= 1.21 × 10** ^**-5**^ **ρ = 1.0 × 10** ^**3**^ **Pe = 4.00 × 10** ^**3**^ **r = 4.3 × 10** ^**-3**^ **Q = 5 × 10** ^**-6**^ **.**

In order to determine the effect of longitudinal tension on the internal pressure, other parameters were held constant while the longitudinal tension was varied. This resulted to three curves that were plotted on the same axes as shown in Figure 3.

From Figure 3 it is observed that as the longitudinal tension increases the internal pressure increases. When the longitudinal tension is 400 N the internal pressure is 3679 Pascals and when the longitudinal tension is increased to 800 N, the internal pressure increases to 3992 Pascals. The increase in internal pressure as the longitudinal tension increases is due to the decrease in flow velocity. From Bernoulli principle, the sum of pressure energy at any part plus the kinetic energy per unit volume plus the potential energy per unit volume at that point is always constant and therefore a decrease in flow velocity leads to an increase in pressure.

#### 2.1.4 Effect of varying volumetric flow rate on the cross sectional area

**Figure 4:**
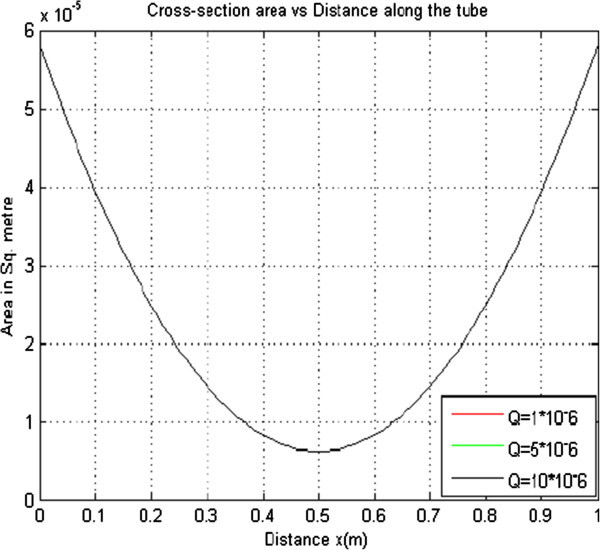
**Cross sectional area versus distance for T = 4.0 × 10** ^**2**^ **K**_**PE**_ **= 1.21 × 10** ^**-5**^ **ρ = 1.0 × 10** ^**3**^ **Pe = 4.00 × 10** ^**3**^ **r = 4.3 × 10** ^**-3**^ **.**

To investigate the effects of varying volumetric flow rate on the cross sectional area of a collapsible tube, the other parameters were held constant while the volumetric flow rate was varied. The curves obtained were plotted on the same axes as shown in Figure 4 below.

From Figure 4 it is observed that change in the volumetric flow rate does not affect the cross sectional area meaning that the cross sectional area is largely independent of the flow rate. The cross sectional area remains as 6.206 × 10^-6^ m/s as the volumetric flow rate changes. This is because as the flow rate increases, the pressure drop increases in order to maintain the steady flow rate.

#### 2.1.5 Effect of varying volumetric flow rate on the flow velocity

**Figure 5:**
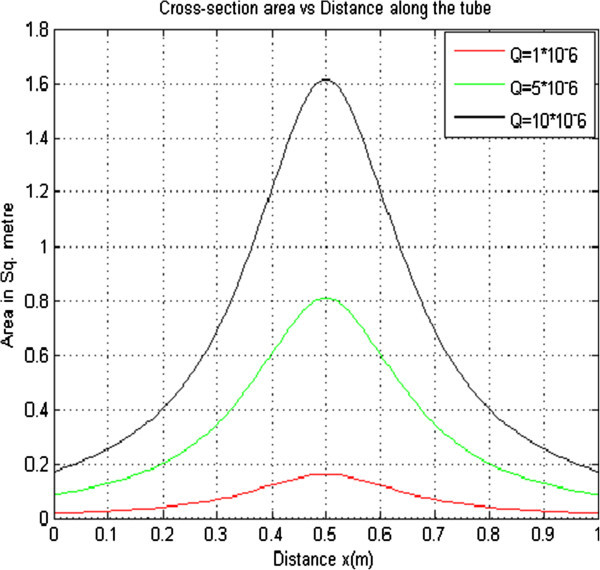
**Flow velocity versus distance for T = 4.0 × 10** ^**2**^ **Kp**_**E**_ **= 1.21 × 10** ^**-5**^ **ρ = 1.0 × 10** ^**3**^ **Pe = 4.00 × 10** ^**3**^ **r = 4.3 × 10** ^**-3**^ **.**

The volumetric flow rate was varied while the other parameters were held constant. The curves obtained were then plotted on the same axes as shown in Figure 5 below.

From Figure 5 it is observed that as volumetric flow rate increases, the flow velocity also increases. When the volumetric flow rate increases from 1 × 10^-6^ to 10 × 10^-6^, the flow velocity increases from 0.1611 m/s to 1.611 m/s. This is because the volumetric flow rate is directly proportional to the flow velocity for a given cross sectional area. In this case the cross sectional area is constant hence an increase in volumetric flow rate translates to an increase in the flow velocity.

#### 2.1.6 Effects of varying volumetric flow rate on the internal pressure

**Figure 6:**
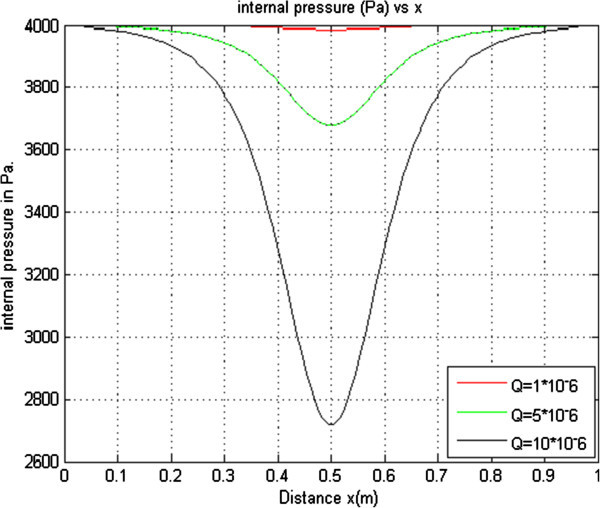
**Internal pressure versus distance for T = 4.0 × 10** ^**2**^ **Kp**_**E**_ **= 1.21 × 10** ^**-5**^ **ρ = 1.0 × 10** ^**3**^ **Pe = 4.00 × 10** ^**3**^ **r = 4.3 × 10** ^**-3**^ **.**

To investigate the effects of varying the volumetric flow rate on the internal pressure of a collapsible tube, different values of volumetric flow rate were used to plot curves while the other parameters were held constant. The curves obtained were plotted on the same axes as shown in Figure 6 below.

From Figure 6, it is noted that as volumetric flow rate increases, the internal pressure decreases. As the volumetric flow rate increases from 1 × 10^-6^ to 10 × 10^-6^, the internal pressure decreases from 3987 Pascals to 2718 Pascals. This is as a result of the already increased flow velocity. From Bernoulli principle an increase in flow velocity leads to a decrease in pressure.

### 2.2 Discussion

The objective of this study was to do an analysis of the flow parameters of a Newtonian fluid through a cylindrical collapsible tube. The flow parameters considered are longitudinal tension and volumetric flow rate. The effects of these flow parameters on the cross sectional area of a collapsible tube, flow velocity and internal pressure have been analyzed.

Longitudinal tension describes how tight the tube is pulled out when attached at the edges while the volumetric flow rate refers to the volume of fluid which passes through a given surface per unit time. The values of the cross sectional area, flow velocity and internal pressure are taken at the point where the collapse of the tube is mostly felt, that being midway along the length of the tube. As fluid flows through the elastic tube there is collision between molecules hence a decrease in kinetic energy. Pressure energy is converted into kinetic energy to maintain the flow velocity since the cross sectional area is the same. This leads to a decrease in internal pressure. Since the external pressure remains constant, it exceeds the internal pressure causing the tube to collapse. The collapse leads to a decrease in cross sectional area and consequently the flow velocity increases in order to maintain a constant flow rate. An increase in velocity of the fluid leads to an increase in the collision between the molecules hence greater loss in kinetic energy. This causes the internal pressure to decrease even more. In addition, according to Bernoulli principle, an increase in fluid velocity leads to a decrease in pressure.

### 2.3 Conclusion

The extent to which the tube collapses is dependent on the longitudinal tension. An increase in this parameter reduces the tube’s tendency to collapse and therefore leads to an increase in the cross sectional area of the tube. Consequently, the flow velocity decreases and the internal pressure increases. It is therefore noted that longitudinal tension is directly proportional to both the cross sectional area and internal pressure and inversely proportional to the flow velocity.

The volumetric flow rate is largely independent of the cross sectional area and therefore any change in the discharge affects the flow velocity and consequently the internal pressure. The volumetric flow rate is directly proportional to the flow velocity and inversely proportional to the internal pressure.

## Nomenclature

**Symbol, Meaning**

T, Longitudinal tension, N

P, Internal pressure, Pa

P_E_, External pressure, Pa

P_t,_ Transmural pressure (P-P_E_), Pa

*u*, Fluid velocity, *ms*^-1^

Q, Volumetric flow rate, *m*^3^*s’*^-1^

A, Cross sectional area, *m*^*2*^

A_0_, Area at the inlet, *m*^*2*^

S, Peripheral length, *m*

K_PE_, Tube stiffness, Pa

F_l_, Skin friction co-efficient

**Greek symbol**

*υ*, Kinematic viscocity of the fluid, *m*^2^*s*^-1^

*ρ*, Fluid density, *kgm*^*-3*^

*μ*, Coefficient of viscosity, *kgm*^*-1*^ *s*^*-1*^
